# Supplementary material for: Multiplex genomic tagging of mammalian ATG8s to study autophagy
Source: J Biol Chem. 2024 Oct 19;300(12):107908. doi: 10.1016/j.jbc.2024.107908 (PMC11607642; doi:10.1016/j.jbc.2024.107908)
Supplement: Figure S1 [file mmc1.pdf]

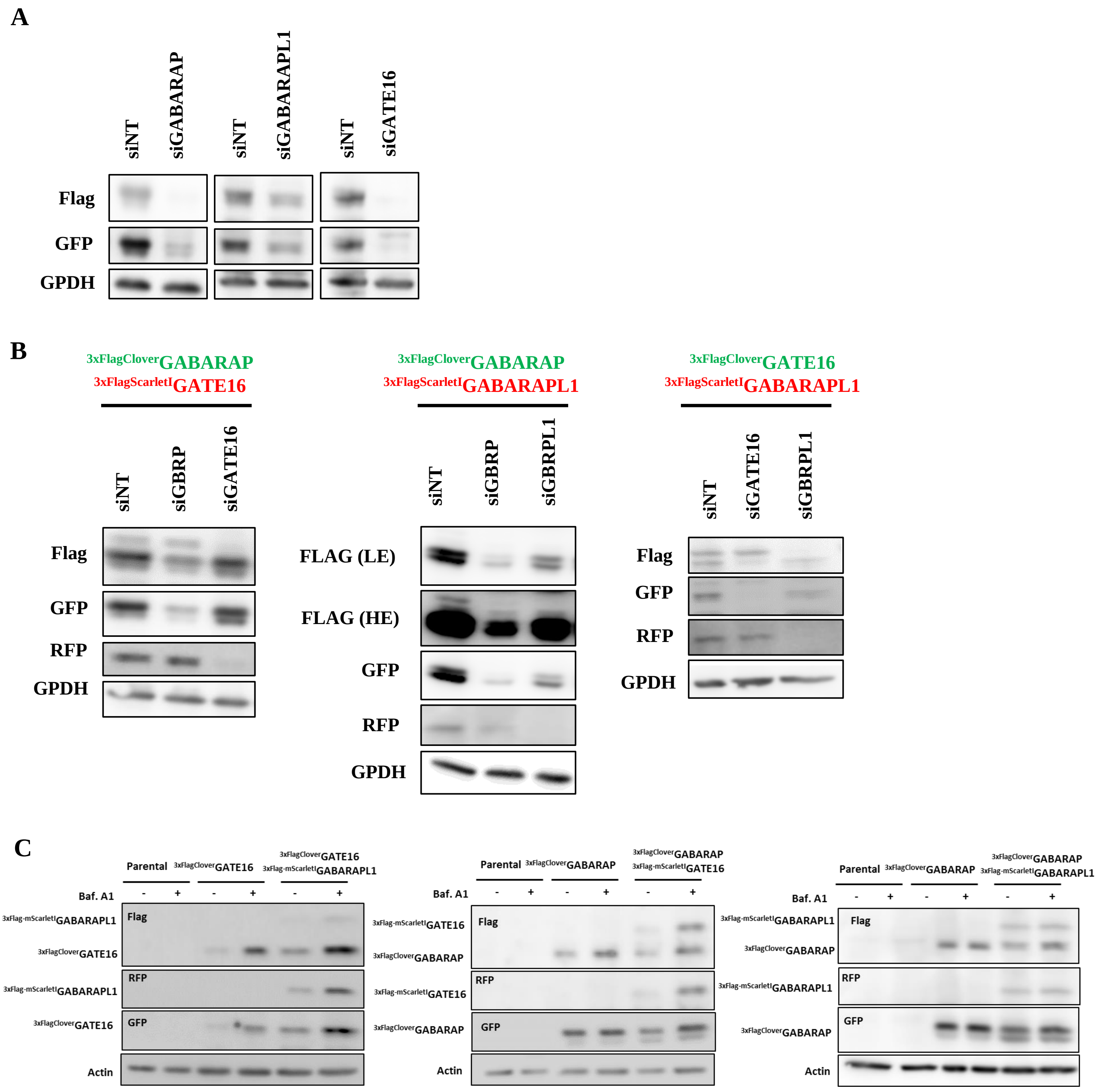

Figure S1: Generation of single and duplex GABARAPs reporter cell lines. A. GABARAPs single color reporter cells were transfected with nontargeting (control) siRNA (si*NT*) or GABARAP or GATE16 or GABARAPL1 siRNA using DharmaFECT1 transfection reagent for 72 h. Total protein extracts were analyzed by western blotting for Flag, GFP, and GPDH (loading control). **B.** GABARAPs duplex reporter cells were transfected with nontargeting (control) siRNA (si*NT*) or with siGABARAP, siGATE16, or siGABARAPL1, using DharmaFECT1 transfection reagent for 72 h. Total protein extracts were analyzed by western blotting for Flag, GFP, RFP, and GPDH (loading control). **C.** Endo-tagged and dual-color endo-tagged GABARAPs expression validated in all reporter cell lines by western blot analysis. The parental *HeLa* cells (control) and GABARAPs reporter cell lines were grown to confluence in a complete medium and treated (where indicated) for the last four hours with 0.1 µM Bafilomycin A1. Total protein extracts were analyzed for Flag, RFP or GFP and actin (loading control).
