# Supplementary material for: Multiplex genomic tagging of mammalian ATG8s to study autophagy
Source: J Biol Chem. 2024 Oct 19;300(12):107908. doi: 10.1016/j.jbc.2024.107908 (PMC11607642; doi:10.1016/j.jbc.2024.107908)
Supplement: Figure S2 [file mmc2.pdf]

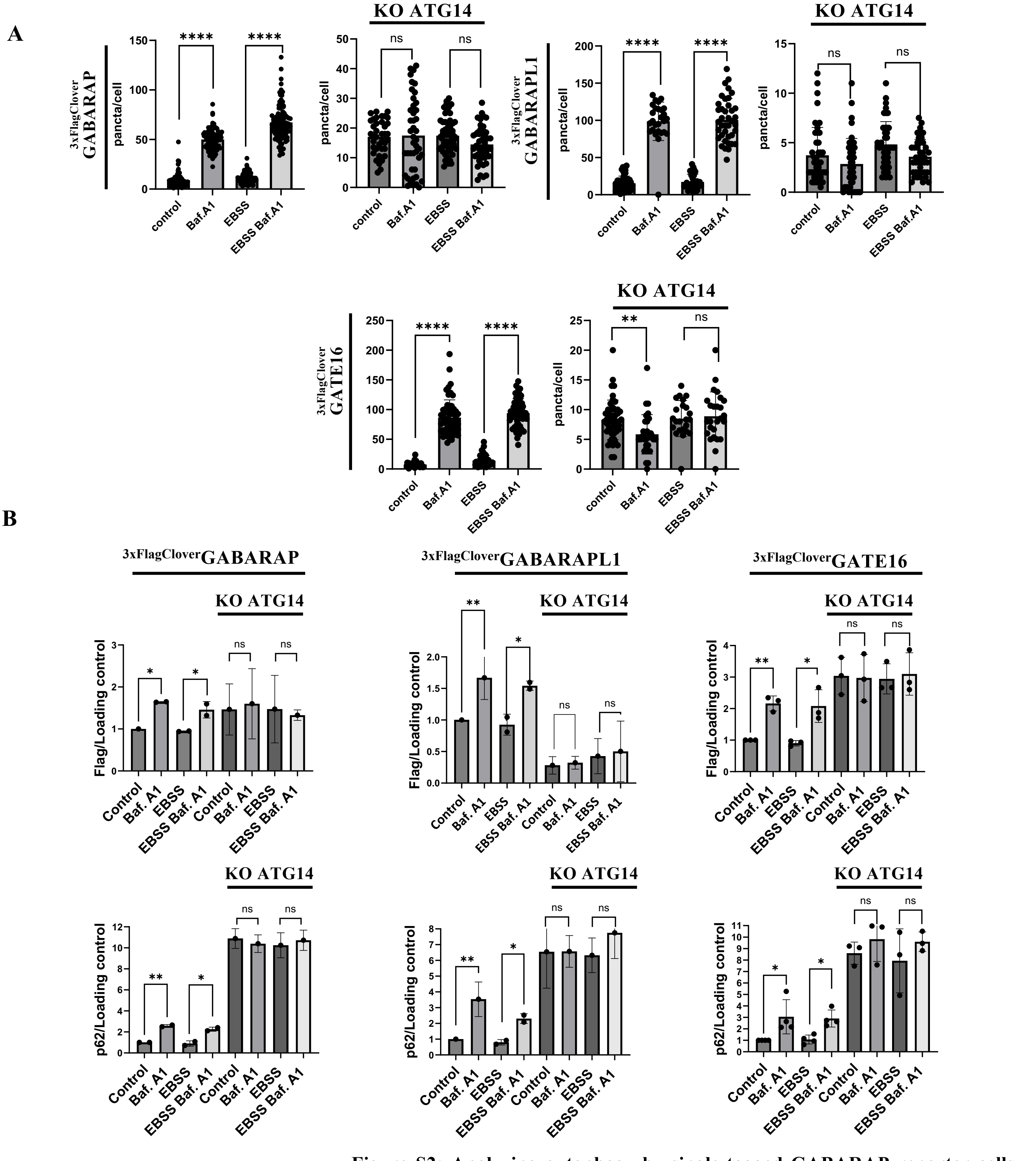

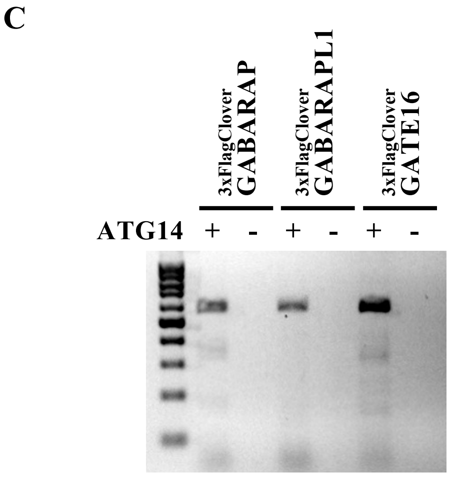

Figure S2: Analyzing autophagy by single-tagged GABARAP reporter cells. **A.** Image analysis of single endo tagged GABARAPs response to autophagy-inducing conditions with and without ATG14 knockout as depicted in *Figure 2A*. For analysis, the visualization was done using a spinning disk confocal. Number of pancta/cell calculated using ImageJ. Images were subjected to maximum projection and background subtraction using the *rolling-ball* and *watershed* functions, quantified using ROIs for single cells. Data are presented with the SEM from three independent experiments, with statistical significance determined by a t-test (****p < 0.0001 and ns- insignificant.). **B.** Western blots quantification of single endo-tagged GABARAPs response to autophagy-inducing conditions with and without ATG14 knockout as depicted in *Figure 2B*. SQSTM1 (p62) and 3xFlag-CloverGABARAPs flux was visualized by p62 and Flag antibodies with the SEM of three independent experiments, *p < 0.05, **p < 0.01 determined by t-test. **C.** Reverse PCR for ATG14 knockout detection. Cells were grown to confluence, followed by total RNA extraction and reverse transcription. cDNA was used as a template for PCR, using primers targeting the ATG14 transcribed region.
