# Supplementary material for: Multiplex genomic tagging of mammalian ATG8s to study autophagy
Source: J Biol Chem. 2024 Oct 19;300(12):107908. doi: 10.1016/j.jbc.2024.107908 (PMC11607642; doi:10.1016/j.jbc.2024.107908)
Supplement: Figure S3 [file mmc3.pdf]

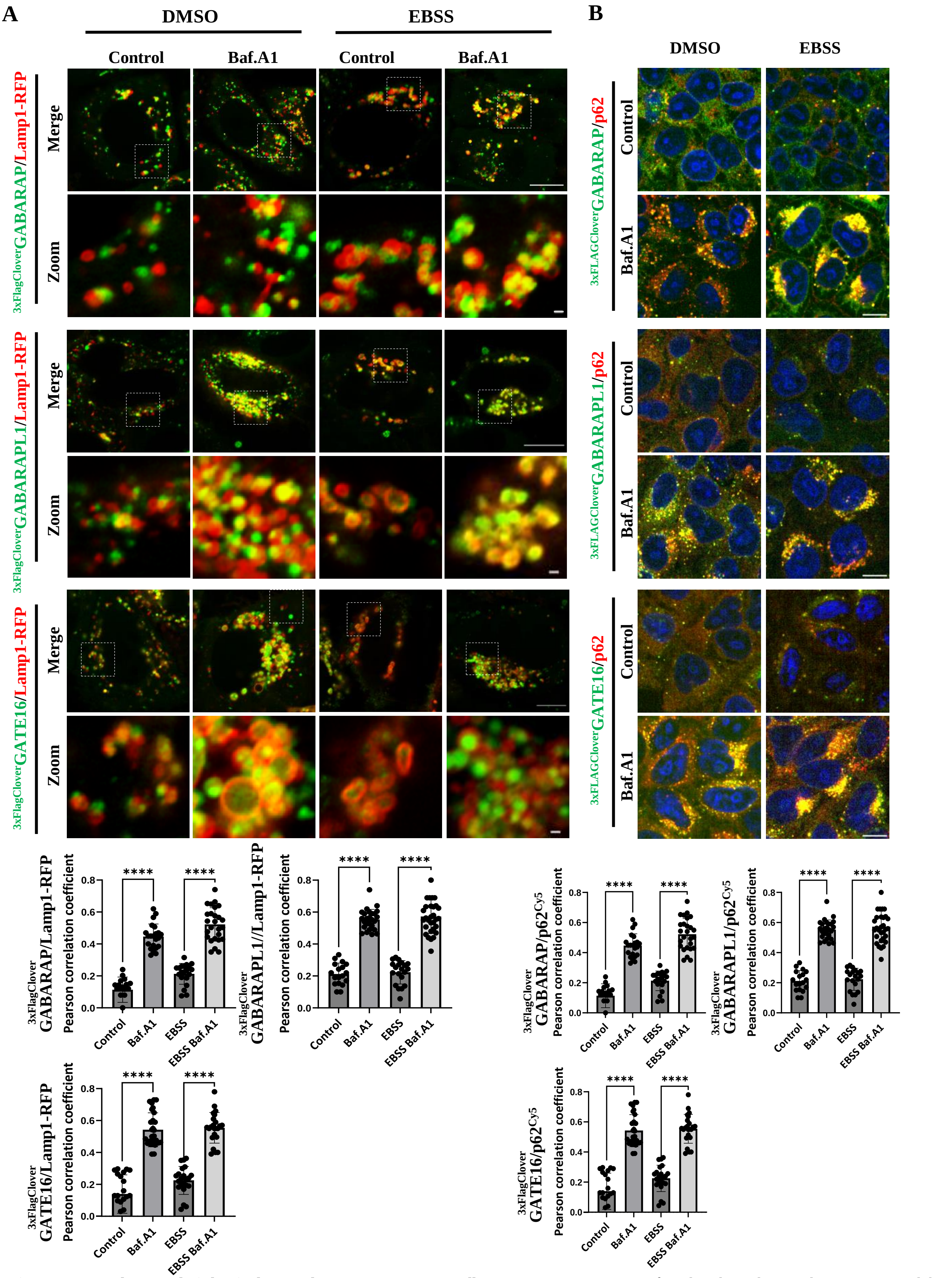

Figure S3: Autophagy analysis by single-tagged GABARAP reporter cells. A. Representative images of single-color endo-tagged GABARAPs and the lysosomal marker LAMP1 by Airyscan super-resolution microscopy. All three reporter cell lines were transfected with the LAMP1-RFP plasmid using JetPrime reagent for 48 hours. Subsequently, cells were incubated for 4 hours in a control medium (DMSO) or starvation medium (EBSS) and treated with 0.1 µM Bafilomycin A1 were indicated for 4 hours. Scale bars: 10µm and 1µm. Colocalization was quantified by Pearson correlation coefficient for LAMP1-RFP and GABARAPs, calculated using ROIs for single cells by *Coloc2* module with 10 *Costes* iterations in ImageJ, and data are presented with the SEM from three independent experiments. Statistical significance was determined by a *t*-test, with ****p < 0.0001. **B.** Single color endo-tagged GABARAPs cells were incubated in complete medium or EBSS in the presence of 0.1 µM Bafilomycin A1 where indicated, for 4 h. Then cells were fixed in absolute methanol and immunostained with SQSTM1 (p62) antibody. The visualization was performed using spinning disk confocal microscopy. Scale bar is 10µm. Colocalization was quantified using ROIs for single cells, by Pearson correlation coefficient for SQSTM1 (p62) and GABARAPs, using *Coloc2* module with 10 costers iterations at ImageJ and presented with the SEM of three independent experiments, ****p < 0.0001 determined by *t*-test.
