# Supplementary material for: Multiplex genomic tagging of mammalian ATG8s to study autophagy
Source: J Biol Chem. 2024 Oct 19;300(12):107908. doi: 10.1016/j.jbc.2024.107908 (PMC11607642; doi:10.1016/j.jbc.2024.107908)
Supplement: Figure S4 [file mmc4.pdf]

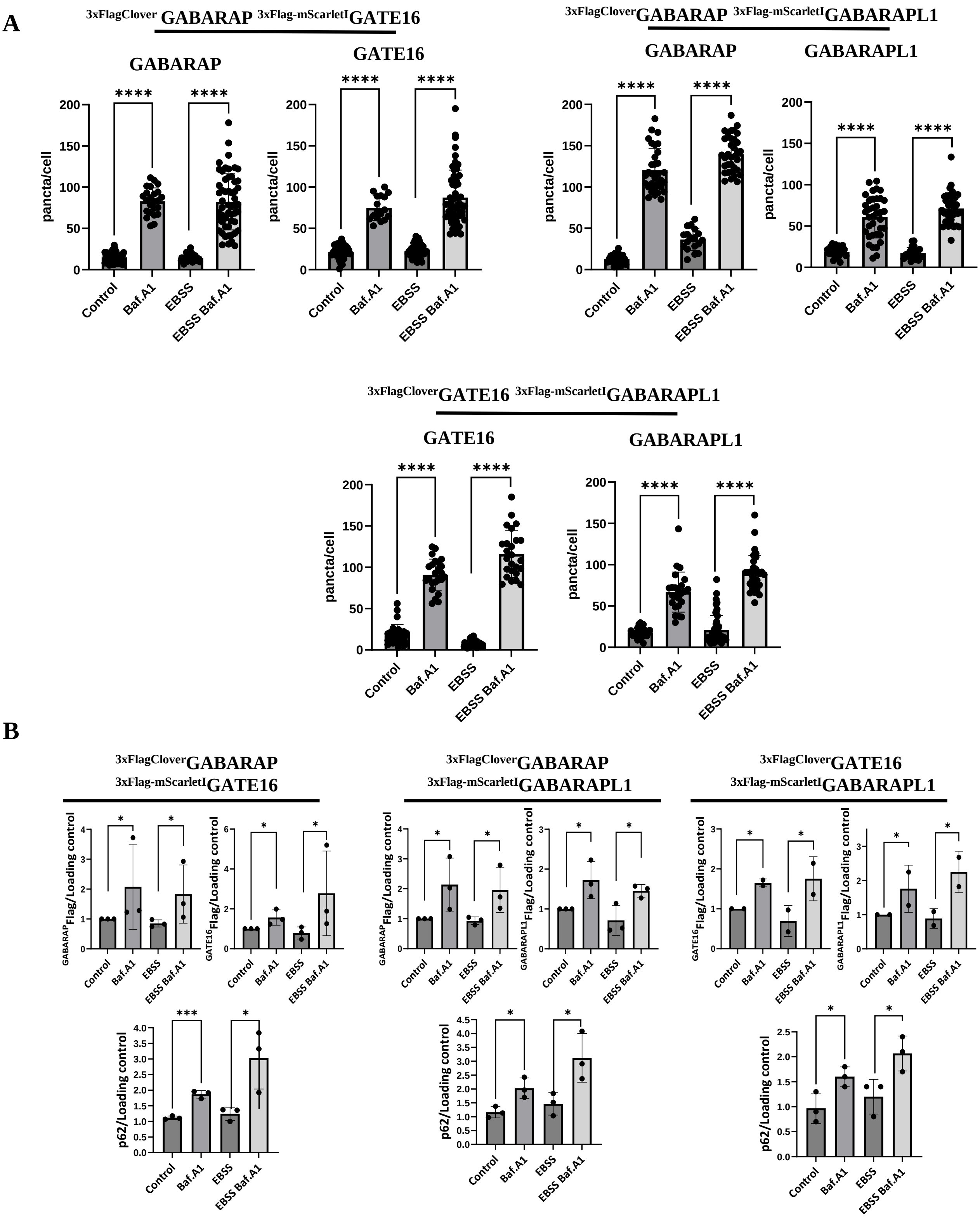

Figure S4: Measuring autophagy with the duplex GABARAPs reporter system. A. Image analysis of duplex endo-tagged GABARAPs under basal and starvation conditions. as depicted in *Figure 3A*. For analysis, the visualization was done using a spinning disk confocal. Number of pancta/cell for each channel calculated using ImageJ. Images were subjected to maximum projection and background subtraction using the *rolling-ball* and *watershed* functions, quantified using ROIs for single cells. Data are presented with the SEM from three independent experiments, with statistical significance determined by *t*-test (***$p < 0.001$, ****$p < 0.0001$). **B.** Duplex endo-tagged GABARAPs response to autophagy-inducing conditions indicated by western blot analysis as depicted in *Figure 3B*. Cells were grown to confluence in a complete and starvation (EBSS) medium and treated (where indicated) for the last 4 h with 0.1 μM Bafilomycin A1. Total protein extracts were probed for Flag, SQSTM1 (p62), GFP, RFP and GPDH (loading control). Quantifications of protein levels for Flag and SQSTM1 (p62) with SEM of three independent experiments, *$p < 0.05$, were determined by *t*-test.
