# Supplementary figures and images for: Multiplex genomic tagging of mammalian ATG8s to study autophagy
Source: J Biol Chem. 2024 Oct 19;300(12):107908. doi: 10.1016/j.jbc.2024.107908 (PMC11607642; doi:10.1016/j.jbc.2024.107908)

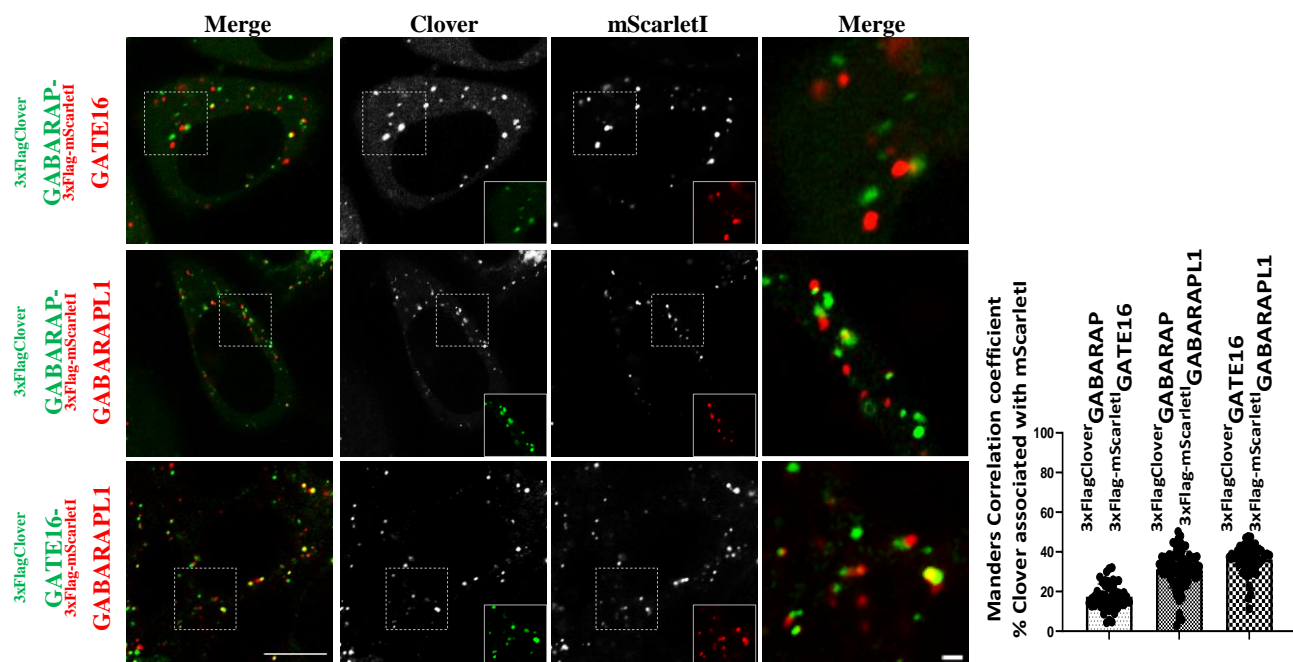

Supplement: Figure S5 [file mmc5.pdf]
