# Supplementary material for: Multiplex genomic tagging of mammalian ATG8s to study autophagy
Source: J Biol Chem. 2024 Oct 19;300(12):107908. doi: 10.1016/j.jbc.2024.107908 (PMC11607642; doi:10.1016/j.jbc.2024.107908)
Supplement: Figure S6 [file mmc6.pdf]

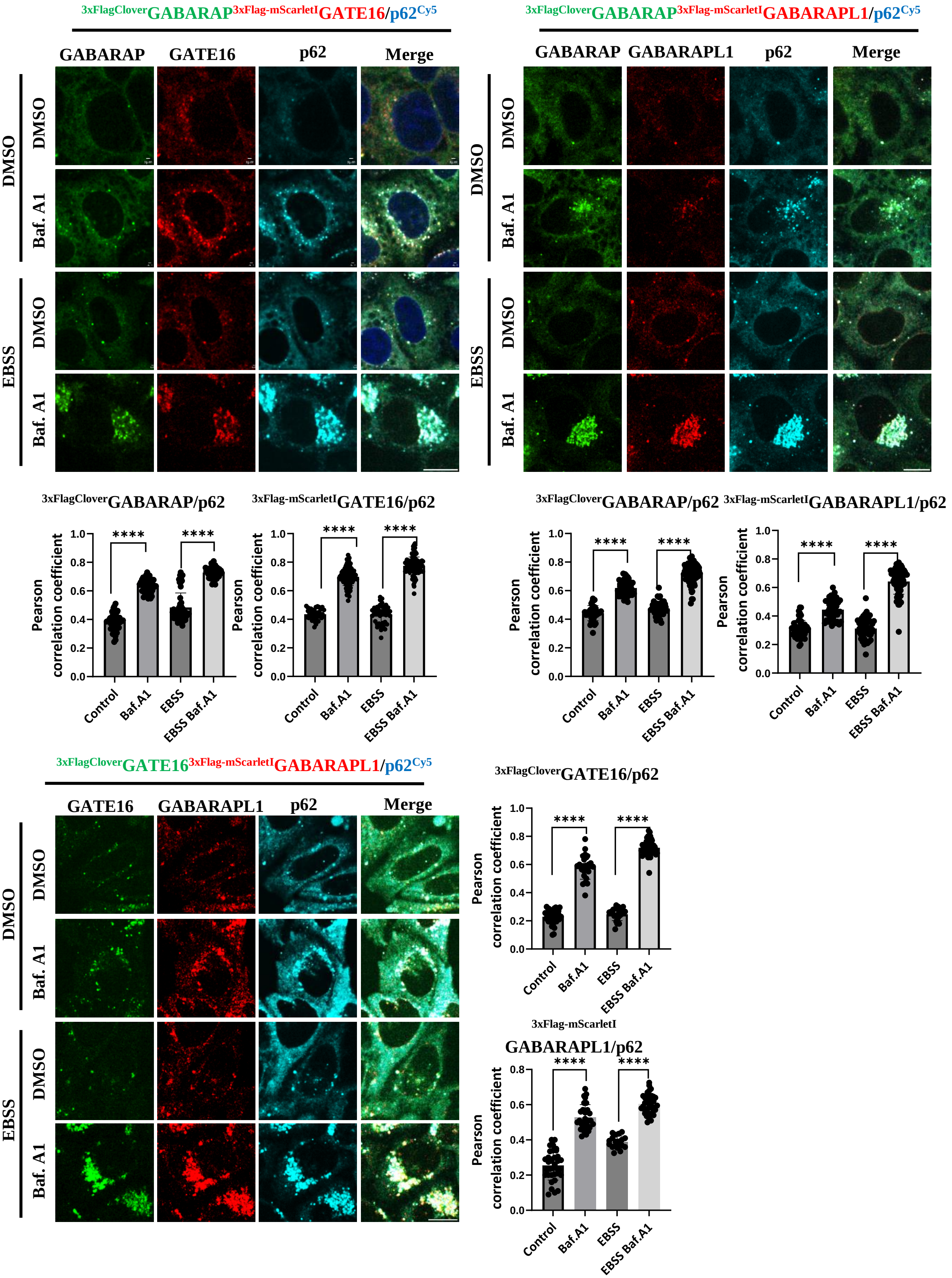

Figure S6: Measuring starvation induced autophagy with the duplex GABARAPs reporter system. Duplex GABARAPs reporter cell lines were incubated in complete medium or EBSS in the presence of 0.1 µM Bafilomycin A1 where indicated, for 4 h. Then cells were fixed in absolute methanol and immunostained with SQSTM1 (p62) antibody. The visualization was performed using spinning disk confocal microscopy. Scale bar 10µm. Colocalization was quantified using Pearson correlation coefficient for p62 and GABARAPs, employing the *Coloc2* module with 10 Costes iterations in ImageJ, using ROIs for single cell. Data from three independent experiments are presented with the SEM. Statistical significance was determined by a *t*-test, with ***$p < 0.001$, ****$p < 0.0001$.
