# Supplementary material for: Multiplex genomic tagging of mammalian ATG8s to study autophagy
Source: J Biol Chem. 2024 Oct 19;300(12):107908. doi: 10.1016/j.jbc.2024.107908 (PMC11607642; doi:10.1016/j.jbc.2024.107908)
Supplement: Figure S7 [file mmc7.pdf]

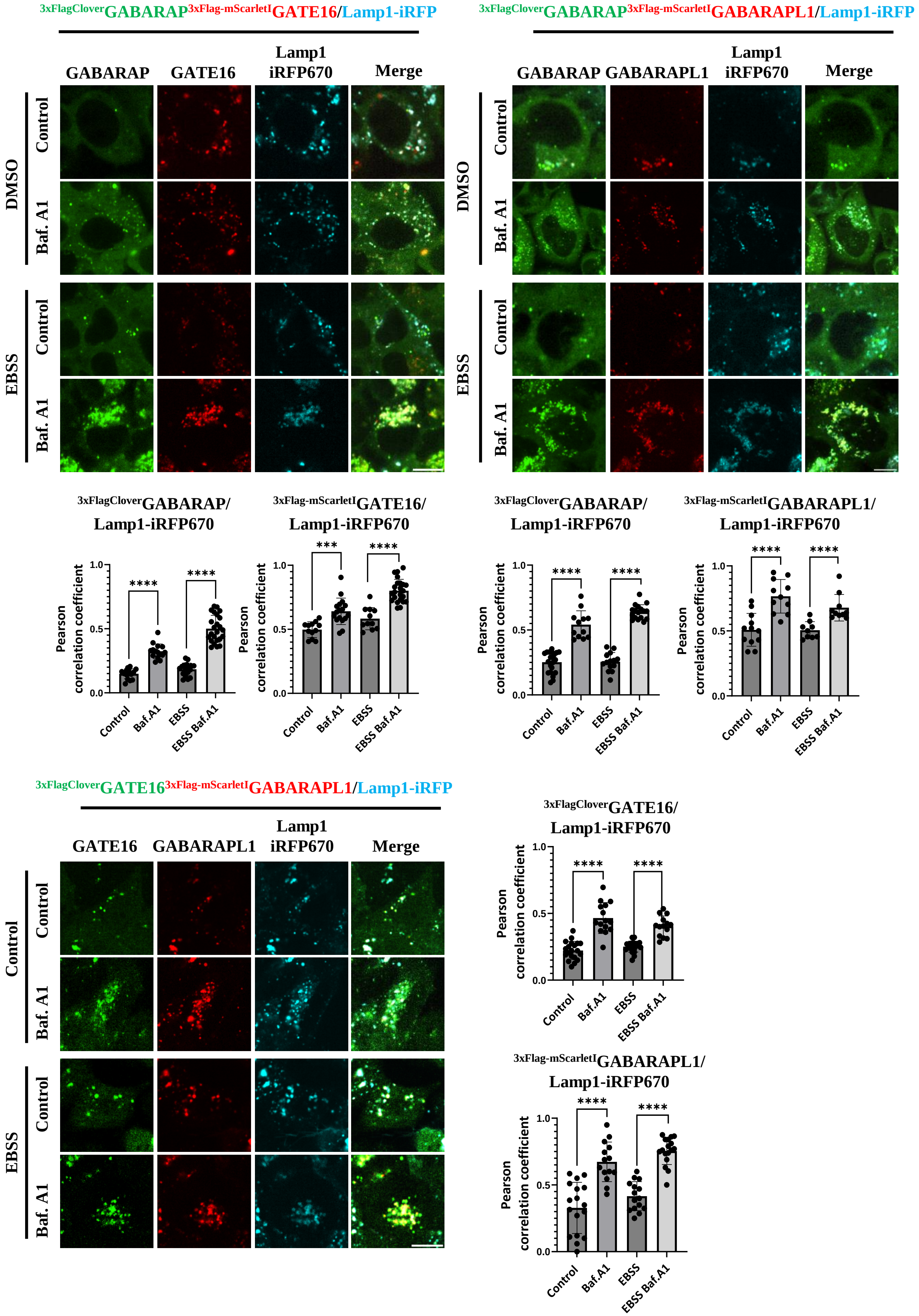

Figure S7: Measuring starvation induced autophagy with the duplex GABARAPs reporter system. Duplex GABARAPs reporter cells exogenously expressing Lamp1-iRPF670 were incubated in complete medium or EBSS in the presence of 0.1 µM Bafilomycin A1, where indicated, for 4 h. The visualization was performed using spinning disk confocal microscopy. Scale bar 10µm. Colocalization was quantified using the Pearson correlation coefficient for iRFP670 and Clover/mScarlet puncta, employing the *Coloc2* module with 10 Costes iterations in ImageJ, using ROIs for single cells. Data are presented with the SEM from three independent experiments. Statistical significance was determined by a *t*-test, with ****p < 0.0001.
