# Supplementary material for: Multiplex genomic tagging of mammalian ATG8s to study autophagy
Source: J Biol Chem. 2024 Oct 19;300(12):107908. doi: 10.1016/j.jbc.2024.107908 (PMC11607642; doi:10.1016/j.jbc.2024.107908)
Supplement: Figure S8 [file mmc8.pdf]

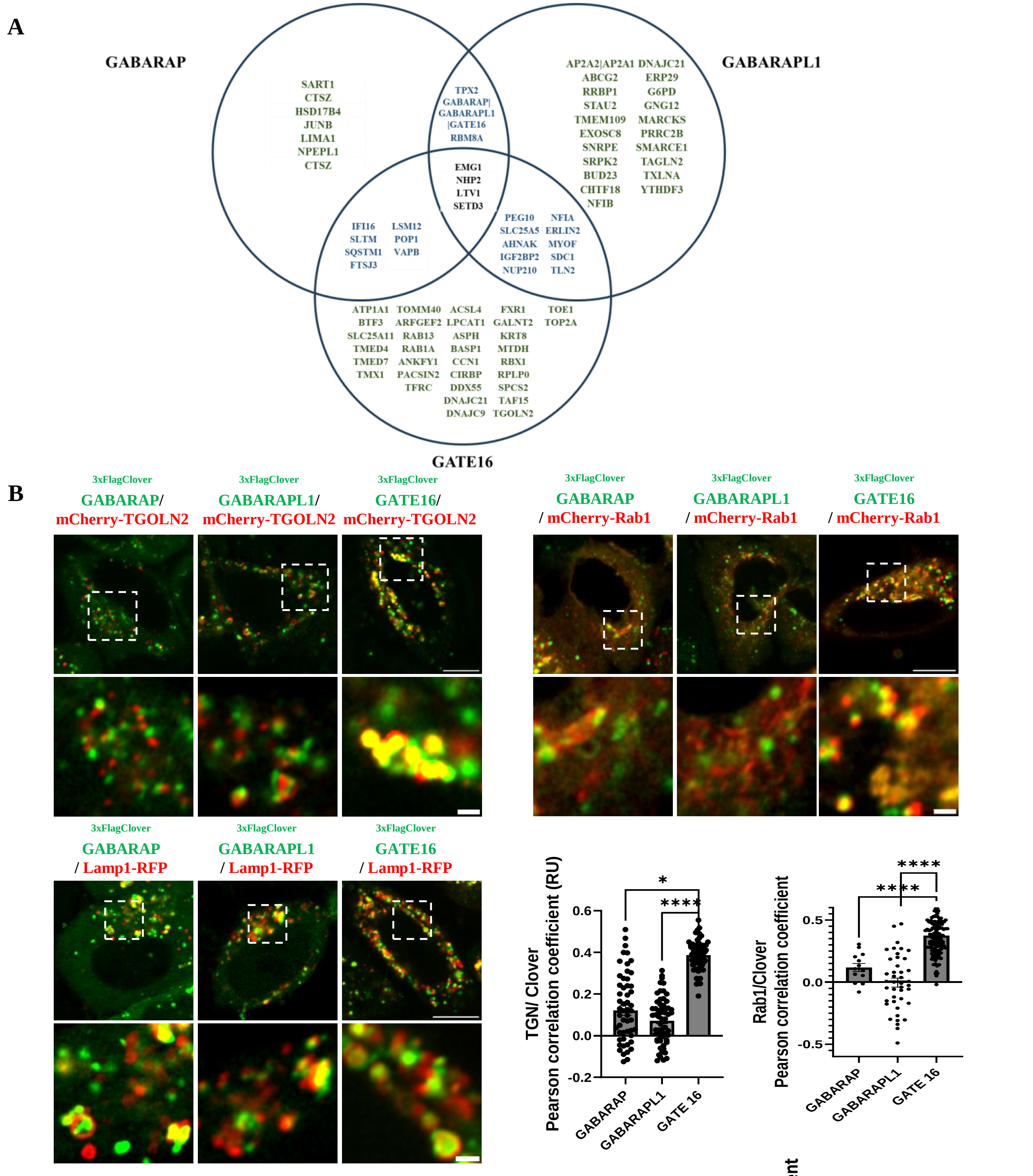

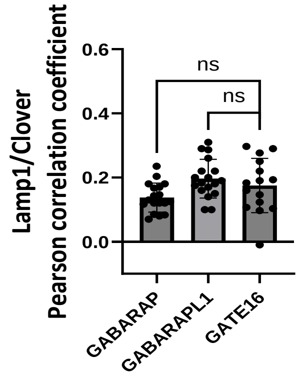

Figure S8: Isolation of distinct autophagic vesicles from endo-tagged GABARAPs cells. A. Venn diagram of overlapping proteins hits found between and among GABARAP subfamilies as determined by LC-MS/MS. Venn diagram was created using the online server Venny 2.1.0 (An interactive tool for comparing lists with Venn's diagrams. (https://bioinfogp.cnb.csic.es/tools/venny/index.html). Protein hits are indicated by consensus gene names in the diagram. **B.** Representative images of single-color endo-tagged GABARAPs transfected with mCherry-TGOLN2, mCherry-Rab1 and Lamp1-RFP plasmids by Airyscan super-resolution microscopy. All three reporter cell lines were transfected using JetPrime reagent for 48 hours. Scale bars are 10µm and 1µm. For analysis, the visualization was performed using scanning confocal microscopy. Colocalization was quantified by Pearson correlation coefficient for mCherry-TGOLN2, mCherry-Rab1 and Lamp1-RFP and GABARAPs, calculated using ROIs for single cells by *Coloc2* module with 10 *Costes* iterations in ImageJ, and data are presented with the SEM from three independent experiments. Statistical significance was determined by a t-test, with $^{*}p < 0.05$, $^{****}p < 0.0001$, ns - non-significant.
