# Supplementary material for: Multiplex genomic tagging of mammalian ATG8s to study autophagy
Source: J Biol Chem. 2024 Oct 19;300(12):107908. doi: 10.1016/j.jbc.2024.107908 (PMC11607642; doi:10.1016/j.jbc.2024.107908)
Supplement: Table S1 [file mmc9.pdf]

Table S1 Antibodies and sequences

**Antibodies**	**Company**	**Species**	**Catalog number**
SQSTM1	BioRad	mouse	2C11
GFP	Invitrogen	rabbit	A-6455
RFP	ChromoTek	mouse	6g6-100
GAPDH	Merck	mouse	MAB374
GABARAP	MBL	rabbit	PM037
GABARAPL1	bioss	rabbit	BS8035R
GABARAPL2	MBL	rabbit	PM038
LC3B	Homemade	rabbit	
FLAG	Sigma-Aldrich	mouse	F1804
Anti-FLAG® M2 Magnetic Beads	Sigma-Aldrich	mouse	M8823

Endogenous tagging cassette

**Knock-in cassette**	**Sequence**
3XFlag-mScarletI-$(GGGS)_3$	gactataaggaccacgacggagactacaaggatcatgatattgattacaaagacgatgacgataagGTGAGCAAGGGCGAGGCAGTGATCAAGGAGTTCATGCGGTTCAAGGTGCACATGGAGGGCTCCATGAACGGCCACGAGTTCGAGATCGAGGGCGAGGGCGAGGGCCGCCCCTACGAGGGCACCCAGACCGCCAAGCTGAAGGTGACCAAGGGTGGCCCCCTGCCCTTCTCCTGGGACATCCTGTCCCCTCAGTTCATGTACGGCTCCAGGGCCTTCATCAAGCACCCCGCCGACATCCCCGACTACTATAAGCAGTCCTTCCCCGAGGGCTTCAAGTGGGAGCGCGTGATGAACTTCGAGGACGGCGGCGCCGTGACCGTGACCCAGGACACCTCCCTGGAGGACGGCACCCTGATCTACAAGGTGAAGCTCCGCGGCACCAACTTCCCTCCTGACGGCCCCGTAATGCAGAAGAAGACAATGGGCTGGGAAGCGTCCACCGAGCGGTTGTACCCCGAGGACGGCGTGCTGAAGGGCGACATTAAGATGGCCCTGCGCCTGAAGGACGGCGGCCGCTACCTGGCGGACTTCAAGACCACCTACAAGGCCAAGAAGCCCGTGCAGATGCCCGGCGCCTACAACGTCGACCGCAAGTTGGACATCACCTCCCACAACGAGGACTACACCGTGGTGGAACAGTACGAACGCTCCGAGGGCCGCCACTCCACCGGCGGCATGGACGAGCTGTACAAG**ggcgggggagtgggggaggatccggaggcggttcc**
3XFlag-Clover-$(GGGS)_3$	gactataaggaccacgacggagactacaaggatcatgatattgattacaaagacgatgacgataagGTGAGCAAGGGCGAGGAGCTGTTCACCGGGGTGGTGCCCATCCTGGTCGAGCTGGACGGCGACGTAAACGGCCACAAGTTCAGCGTCCGCGGCGAGGGCGAGGGCGATGCCACCAACGGCAAGCTGACCCTGAAGTTCATCTGCACCACCGGCAAGCTGCCCGTGCCCTGGCCCACCCTCGTGACCACCTTCGGCTACGGCGTGGCCTGCTTCAGCCGCTACCCCGACCACATGAAGCAGCACGACTTCTTCAAGTCCGCCATGCCCGAAGGCTACGTCCAGGAGCGCACCATCTCTTTCAAGGACGACGGTACCTACAAGACCCGCGCCGAGGTGAAGTTCGAGGGCGACACCCTGGTGAACCGCATCGAGCTGAAGGGCATCGACTTCAAGGAGGACGGCAACATCCTGGGGCACAAGCTGGAGTACAACTTCAACAGCCACAACGTCTATATCACGGCCGACAAGCAGAAGAACGGCATCAAGGCTAACTTCAAGATCCGCCACAACGTTGAGGACGGCAGCGTGCAGCTCGCCGACCACTACCAGCAGAACACCCCCATCGGCGACGGCCCCGTGCTGCTGCCCGACAACCACTACCTGAGCCATCAGTCCGCCCTGAGCAAAGACCCCAACGAGAAGCGCGATCACATGGTCCTGCTGGAGTTCGTGACCGCCGCCGGGATTACACATGGCATGGACGAGCTGTACAAG**ggcgggggagtgggggaggatccggaggcggttcc**

gRNAs list

**Gene Symbol**	**CRISPR gRNA**		**Mutated PAM site at the homologous arm in the donor plasmid**

GABARAP	GTTCGAGAAGCGCCG CTCTGAGG	(+)	AAG
GABARAPL1	CATGAAGTTCCAGTA CAAGGAGG	(+)	AAG
GABARAPL2	TTCAAGGAGGACCAC TCGCTGGG	(-)	GGA
ATG14	CTACTTCGACGGCCG CGACCGGG	(+)	-

Genotyping primers used for sequencing analysis of the knockout lines

**Gene**	**Exon**	**Primer Direction**	**Primer sequence (5′ to 3′)**	**PCR product (bp)**
GABARAP	1	Forward	TTGGTGAATAGGG AAGTGGCGC	wt 591<br>kI 1407
		Reverse	CCCAATAGGGCGT CACCATAA	
GABARAPL1	1	Forward	CCGGTATTTCTCC ATCTGGCT	wt 640<br>kI 1455
		Reverse	CGATCTAACCAGT CTCGCCC	
GATE16	1	Forward	TTGTGCTCGGTGC GCTG	wt 300<br>KI 1111
		Reverse	ACCACACGCTCTG GAGGTTA	
ATG14	1	Forward	AAAATCCCACGTG ACTGGCT	566
		Reverse	TCCCCTACTAGCT CCCAGCAAG	

Reverse Transcription PCR (RT-PCR)

**Gene**	**Primer Direction**	**Primer sequence (5′ to 3′)**	**PCR product (bp)**
ATG14	Forward	CGCTGTGCAACACTACCCG	443
	Reverse	TGCCAGACGCTCATAATGACT	
